# Study of the erythropoiesis activity of nano-encapsulated forms of erythropoietin

**DOI:** 10.5195/cajgh.2013.110

**Published:** 2014-01-24

**Authors:** Zhanagul Khasenbekova, Alexandr Gulayev, Elena Nechayeva, Talgat Nurgozhin, Zhaxybay Zhumadilov

**Affiliations:** 1 Center for Life Sciences, Nazarbayev University; 2 The State Research Center of Virology and Biotechnology VECTOR, Novosibirsk, Russia

**Keywords:** erythropoiesis, nanobiotechnology, recombinant human erythropoietin

## Abstract

**Introduction:**

The recombinant human erythropoietin (rhEPO) is used in the treatment of anemia. In order to improve its pharmacokinetic properties, nanoparticles of biodegradable polymers of natural or synthetic origin were used. The aim of this study was to investigate the effect of new nano-encapsulated forms of recombinant human erythropoietin for oral use on the erythropoiesis in the cyclophosphamide immunosuppression model.

**Material and methods:**

The CHOpE immortalized cells culture (a primary producer of rhEPO “Vector” in Russia) was used. The following biodegradable polymers were chosen: 0.05% and 0.005% carbopol, 0.05% and 0.005% kollidon, and 0.05% and 0.005% pectin. Immunosuppression was obtained by a single dose of i.p. injection of cyclophosphamide (250 mg/kg) in white mice (18–20 g). During the next 5 days, the nano-encapsulated erythropoietin (100 ED/mouse) was administered orally to each mouse. After 5 and 10 days, the cell count of the number of blood reticulocytes and the myelogram of bone marrow were performed. The control group of mice received injections of Eprex.

**Results:**

On the 5th day of the experiment, the highest level of reticulocyte was observed in the samples of erythropoietin with kollidon (0.05%) and pectin (0.005%) nanoparticles. On the 10th day, the highest activity was observed in the samples of erythropoietin substance with pectin at 0.05% and 0.005% concentrations. The levels of reticulocytes in these groups reached 13.53% and 14.55%, respectively. The results of the myelogram during immunosuppression showed some activity of erythropoietin in conjunction with both concentrations of pectin when a two-fold increase in the number of erythroblasts was observed on the 5th day. High degrees of erythrokaryocytes in the state of mitosis were observed in the 0.05% pectin samples. Similar results were observed in equivalent groups of control animals on the 10th day of the experiment, which is compatible with the data on Eprex action.

**Conclusion:**

The erythropoietic activity of nano-encapsulated forms of erythropoietin was observed in the 0.05% and 0.005% pectin samples in the cyclophosphamide immunosuppression model setting.

